# The evaluation of a rapid microfluidic immunofluorescence antigen test in detecting the infectiousness of COVID-19 patients

**DOI:** 10.1186/s12879-023-08821-9

**Published:** 2023-11-23

**Authors:** Kenji Ota, Hina Kodama, Yasuhide Kawamoto, Daisuke Sasaki, Fujiko Mitsumoto-Kaseida, Kei Sakamoto, Kosuke Kosai, Hiroo Hasegawa, Takahiro Takazono, Koichi Izumikawa, Hiroshi Mukae, Mya Myat Ngwe Tun, Kouichi Morita, Katsunori Yanagihara

**Affiliations:** 1 Department of Laboratory Medicine, Nagasaki University Hospital, 1-7-1, Sakamoto, Nagasaki 852-8501 Japan; 2 Department of Microbiology, Graduate School of Medicine, Yamaguchi University, 1-1-1, Minami-Kogushi, Ube, 755-8505 Japan; 3 Department of Respiratory Medicine, Nagasaki University Hospital, 1-7-1, Sakamoto, Nagasaki 852-8501 Japan; 4 Infection Control and Education Center, Nagasaki University Hospital, 1-7-1, Sakamoto, Nagasaki 852-8501 Japan; 5 Department of Virology, Institute of Tropical Medicine (NEKKEN), Nagasaki University, 1-12-4, Sakamoto, Nagasaki 852-8102 Japan; 6 Dejima Infectious Disease Research Alliance, Nagasaki University, 1-12-4, Sakamoto, Nagasaki 852-8102 Japan

**Keywords:** Rapid microfluidic immunofluorescence method, Infectivity, COVID-19, Test-based strategy

## Abstract

**Background:**

A test-based strategy against coronavirus disease 2019 (COVID-19) is one of the measures to assess the need for isolation and prevention of infection. However, testing with high sensitivity methods, such as quantitative RT-PCR, leads to unnecessary isolation, whereas the lateral flow antigen test shows low sensitivity and false negative results. The purpose of this study was to evaluate the performance of the LumiraDx SARS-CoV-2 Ag test (Lumira Ag), a rapid microfluidic immunofluorescence method, in assessing infectivity.

**Methods:**

This study was performed from March 2022 to July 2022. A pair of nasopharyngeal swab samples were obtained from each patient with mild COVID-19. One swab was used for Lumira Ag testing, and the other for quantitative RT-PCR testing and virus culture.

**Results:**

A total of 84 patients were included in the study. Among them, PCR, Lumira Ag test, and virus culture indicated positivity for 82, 66, and 24 patients, respectively. When comparing the Lumira Ag test to virus culture, its sensitivity was 100.0% (24/24), specificity, 30.0% (18/60); positive predictive value, 36.3% (24/66); and negative predictive value (NPV), 100.0% (18/18). The positive sample for virus culture was observed until the ninth day from the onset of symptoms, while the Lumira Ag test was observed until day 11.

**Conclusions:**

The Lumira Ag test showed high sensitivity and NPV (100% each) compared to virus culture. A test-based strategy using the Lumira Ag test can effectively exclude COVID-19 infectiousness.

**Supplementary Information:**

The online version contains supplementary material available at 10.1186/s12879-023-08821-9.

## Background

Due to the continuation of coronavirus disease 2019 (COVID-19) for three years, a uniformly required duration of quarantine for patients is being reconsidered. As an alternative to quarantine, a test-based strategy against COVID-19 is a widely accepted measure to assess the need for isolation and infection prevention [1, 2]. Using this strategy, individuals with negative test results are allowed to end their isolation or remove their masks. For the specific purpose of excluding potential infectiousness, high sensitivity in detecting infectious individuals is an essential characteristic of testing. Furthermore, the infectiousness of the Omicron variant assessed by viral culture has been reported even 5 days after the onset of symptoms [3–5], reinforcing the need for an efficient test-based strategy.

However, nucleic acid amplification testing, including RT-PCR, has a high sensitivity for SARS-CoV-2 regardless of its viability, leading to continuous positive test results for several weeks [6, 7]. Additionally, maintaining a turn-around time of several hours is difficult; therefore, RT-PCR as point-of-care testing is challenging. In contrast, the lateral flow antigen test shows lower sensitivity for viral culture-positive samples [8–10], with potentially false negative results in assessing infectiousness.

The LumiraDx SARS-CoV-2 Ag test (Lumira Ag, LumiraDX UK Ltd., Dumyat, UK) is a rapid antigen test that offers a qualitative test result using a rapid microfluidic immunofluorescence method, which is available as point-of-care testing. Previous studies reported that the Lumira Ag test exhibited higher clinical sensitivity and specificity than RT-PCR [11]. However, the performance of the Lumira Ag test in assessing infectiousness in a test-based strategy is not clear. In this study, we focused on the highly sensitive performance of Lumira Ag testing and aimed to evaluate the performance of the Lumira Ag test for assessing the infectivity of the SARS-CoV-2 Omicron variant.

## Methods

### Sample collection

A prospective study was conducted from March–July 2022 to assess the performance of Lumira Ag in detecting SARS-CoV-2 infectivity. According to the surveillance data, the dominance of Omicron variants was greater than 99.67% during the study period [12]. The study was approved by the Institutional Review Board of Nagasaki University Hospital(Approval Number: 22022126). Patients with COVID-19, diagnosed using nucleic acid amplification or antigen tests and staying in recovery accommodation facilities in Nagasaki city, were enrolled in the study. After informed consent was acquired from patients with mild COVID-19, a pair of nasopharyngeal swab samples were obtained and immediately sent to the laboratory. The swabs were obtained from the same naris by a trained physician. One swab was used for Lumira Ag testing, and the other was stored in virus transport media (VTM) and used for quantitative RT-PCR testing and viral culture.

### Lumira Ag, RT-PCR, and viral culture

Lumira Ag testing was performed according to the instructions of the manufacturer. Briefly, the swab was placed into the extraction buffer, which was applied to the test strip inserted in the LumiraDx instrument. The qualitative result was obtained within 12 min.

Quantitative RT-PCR testing was performed according to the National Institute for Infectious Diseases guidelines [13]. Nucleic acid was extracted and purified from VTM using the MagMAX Viral/Pathogen nucleic acid isolation kit (Thermo Fisher Scientific), following the protocol of the manufacturer. For RT-PCR, 5 μL of the RNA template was tested using real-time RT-PCR primer/probe sets for 2019-nCoV_N2 [13]. PCR was conducted using the Thunderbird probe one-step qRT-PCR kit (TOYOBO) and the QuantStudio 6 Pro real-time PCR system (Thermo Fisher Scientific). The cycle threshold (Ct) value was used to indicate viral samples.

The virus was isolated to check infectivity using swab samples stored in VTM. A 100 µL aliquot of each VTM was inoculated in Vero E6 cells cultured in Eagle's medium supplemented with 2% fetal calf serum and 1% penicillin or streptomycin solution. Infected cells were cultured at 37 °C and observed for cytopathic effects daily. The infected culture fluid (ICF) was collected 5 days after infection for the first passage. The second passage of the viral culture was performed using a fresh monolayer of Vero E6 cells following the same procedure used in the first passage of the virus culture. Approximately 200 µL second passage of ICF was used for viral RNA extraction by the Nextractor NX-48 robot using the NX-48S Viral NA Kit (Genolution Inc.). The presence of the virus in ICF was verified using quantitative real-time RT-PCR. The cytopathic effect in viral culture was assessed and referred to as infectivity [8, 14–16].

### Viral load comparison between the Lumira Ag buffer and VTM as a matrix for PCR testing

To compare the viral load between paired samples and assess the Lumira Ag buffer and VTM as a matrix for the nucleic acid amplification test, the residual buffer from Lumira Ag testing was subsequently used for quantitative RT-PCR testing. The quantitated viral loads were expressed in terms of copies/test and compared on a scatter plot.

### Statistical analysis

Sensitivity, specificity, positive predictive value (PPV), and negative predictive value (NPV) of the Lumira Ag test to viral culture were calculated. The unpaired t-test was performed to compare the viral load between groups. The level of statistical significance was set at *P* < 0.05.

## Results

**Fig. 1:**
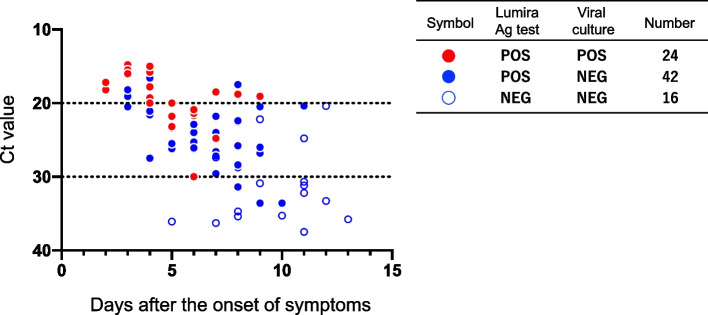
The results of Lumira Ag testing, viral culture, and RT-PCR testing. Qualitative results for Lumira Ag testing and viral culture, and quantitative results for RT-PCR testing (Ct value) are shown. The relationships between the test results and the days after the onset of symptoms are shown. The results of Lumira Ag testing are shown as filled circles for positive results and empty circles for negative results. The viral culture results, representing infectiousness, are shown as red for positive and blue for negative. NEG, negative; POS, positive

A total of 84 patients with COVID-19 (mean age: 42.1 years, male: 54.8%) who were between 2 and 13 days from the onset of symptoms were included in the study. Among them, 82 patients underwent RT-PCR tested positive and subsequently proceeded for further analysis. Lumira Ag showed positive results for 66 of the participants, and viral culture for 24 (Additional file 1). The positive samples for RT-PCR were observed until day 13 from the onset of symptoms, the Lumira Ag test until day 11, and viral culture until day 9 (Fig. 1 and Additional file 2).

The comparison of the Lumira Ag test to viral culture is shown in Table 1. The sensitivity was 100% (24/24), specificity was 27.6% (16/58), PPV was 36.3% (24/66), and NPV was 100% (16/16). No samples were positive in viral culture among the samples with negative results in the Lumira Ag test.

**Table 1:** Comparison of the results of nasopharyngeal swab sample testing by Lumira Ag test and viral culture

		Viral culture		
		POS	NEG	Total
Lumira Ag test	POS	24	42	66
	NEG	0	16	16
	Total	24	58	82

Relative to viral culture, the Lumira Ag test sensitivity was 100% (24/24), specificity was 30% (18/60), positive predictive value was 36.3% (24/66); and negative predictive value was 100% (18/18). NEG, negative; POS, positive.

**Fig. 2:**
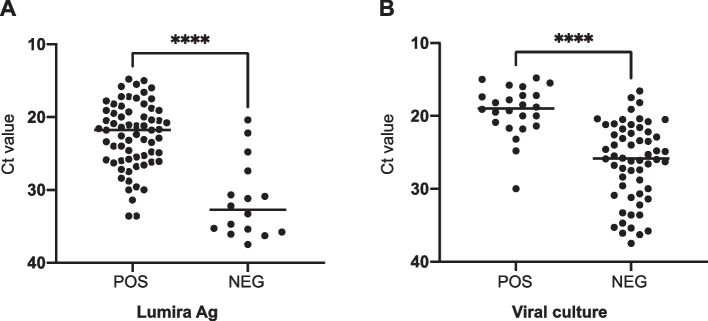
Lumira Ag testing and RT-PCR analysis. **a** Viral load determined using RT-PCR in Lumira Ag-positive and -negative groups. **b** Viral load determined using RT-PCR was compared in viral culture-positive and -negative groups. A *t*-test was applied to assess the differences between groups. ****, *p* < 0.0001; ns, not significant; NEG, negative; POS, positive

When comparing the samples that tested positive by Lumira Ag with those that tested negative, the Ct value determined using RT-PCR was significantly lower in the positive samples (mean, 22.6 vs. 31.5; *P* < 0.0001) (Fig. 2a). Among the samples with a Ct value ≤ 30, the positivity for Lumira Ag was 93.9% (62/66). In addition, a significantly higher Ct value was observed in viral culture-positive samples than in viral culture-negative ones (19.3 vs. 26.4; *P* < 0.0001; Fig. 2b). When the cut-off threshold of the Ct value to viral culture was set at 30.0, the positive predictive value was calculated to be 36.3% (24/66), and the negative predictive value was 100.0% (16/16).

To assess the variation between the paired swabs, the viral load determined by RT-PCR was compared (see Additional file 3), and the correlation coefficient was 0.8136.

## Discussion

In this study, the results of the Lumira Ag test and viral culture were compared and the results were correlated with the viral load determined using RT-PCR. The Lumira Ag test had high sensitivity and NPV relative to viral culture, favoring it as a measure of infectivity.

Nucleic acid amplification testing, including RT-PCR, has been accepted as the gold standard for testing for COVID-19 because of its high sensitivity and specificity. Though almost all clinical research and diagnosis are based on the results of RT-PCR, false-negative results are occasionally observed despite its high analytical sensitivity. A systematic review by Pecoraro et al. [17] reported a wide variation of false-negative rates (2%–58%), and a summary estimate of the overall false-negative rate of 12%. Several possible causes for false-negatives include pre-analytical errors such as quality of clinical specimens [18], sampling procedure, swab type, sample container, as well as the sampling having occurred early in the course of infection, which results in an extremely small amount of virus particles due to the early phase of infection [19]. Thus, clinicians and laboratories need to be aware of the possibility of false-negative results affected by clinical and laboratorial variances, and pre-test probability must be considered in interpreting test results. Another important characteristic of RT-PCR is that it can provide quantitative results based on the cycle threshold (Ct) value, which could be a surrogate of infectivity [20]. However, inter-laboratory variation makes it difficult to apply RT-PCR to assess infectiousness [21]. Additionally, previous studies have reported positive results of RT-PCR several weeks after the diagnosis [22–24]. Multiple samples with a high viral load of > 10^4^/test were observed even 10 days after symptom onset when no culturable virus was detected. Therefore, RT-PCR has limited practicality in predicting infectiousness.

Rapid antigen testing, such as the lateral flow test, is easy to perform and is widely used as point-of-care testing by healthcare and non-healthcare providers. Therefore, the lateral flow test is a representative test intended to be utilized as part of a test-based strategy, particularly where immediate decisions are required. However, it is well proven that the target population who are to be tested has a great impact on the sensitivity of rapid antigen testing. In the Cochrane review [25], the sensitivity was reported to be 73.0% in symptomatic patients and further increased to 80.9% in those exhibiting symptoms in seven days. On the other hand, the sensitivity remains at 54.7% in asymptomatic patients, with a slightly higher sensitivity of 64.3% in those with previous contact with the patients. To compensate for its low sensitivity, repeated use of rapid antigen testing is suggested and the effectiveness of this method is supported by several clinical studies [26, 27]. However, as an assay format, lateral flow assay shows relatively lower sensitivity (61.4% for alkaline phosphatase-labeled antibodies and 81.3% for latex-conjugated) compared to microfluidic fluorescent immunoassay (89.7%) [25]. Accordingly, for the assessment of infectiousness, a previous study by Kirby et al. [8] reported that the sensitivity of the lateral flow test decreased, which may result in a failure to exclude infectious individuals. The Lumira Ag test is reported to have higher sensitivity than the lateral flow test in detecting SARS-CoV-2 [28, 29], and the results of the present study showed its satisfactory performance in effectively excluding infectious COVID-19 patients. Based on the high NPV of Lumira Ag to viral culture, the exclusion of infectivity in patients with COVID-19 can be proposed as a clinical application. Time-based strategies, which require uniform durations of isolation (e.g. five to ten days), are no longer a practical measure. However, as shown in this study, some patients exhibited infectivity even after five days after the onset of the symptom. To compensate in test-based strategies, highly sensitive diagnostic testing such as Lumira Ag can play an important role in excluding infectiousness and help shorten the isolation period. Since this requires the instrument for testing and must be performed in medical facilities, this application of the use of Lumira Ag testing is suitable for medical staff. By applying this test-based strategy, the isolation period can be shortened and the risk of spreading the infection to the patient and other staff can be minimized. Also, Lumira Ag showed satisfactory performance as a diagnostic test with a positivity of 93.9% for samples with a Ct value ≤ 30 (62/66). Consequently, it is an alternative testing device with acceptable sensitivity in clinical settings where nucleic acid amplification tests cannot easily be deployed because of time or manpower constraints.

However, the low specificity for infectiousness observed in this study (30%) raises concerns that positive results of Lumira Ag testing should not be interpreted as evidence for infectivity. The sample with a positive result of Lumira Ag testing was observed on the 11^th^ day after the onset of symptoms and contained no culturable virus.

Viral culture reflects the replication-competent viral particles contained in a clinical specimen and is accepted as a measure to assess infectiousness. Previous clinical studies have defined viral culture as an indicator of infectiousness [16, 30]. However, since viral culture takes several days to report results and requires biosafety level 3 precautions, it is not common to perform viral culture tests in clinical laboratories for the purpose of clinical assessment of infectiousness. The difficulties inherent to viral culture even raise the importance of the evaluation of clinically available testing for assessing infectiousness. Indicators for viral culture include some difficult clinical cases under immunocompromised status with continuous positive viral shedding for weeks and months, when the results of viral culture aid clinical decisions regarding discontinuation of antiviral drug regimens or isolation [21, 22].

The limitations of this study include that the Lumira Ag testing and other tests were performed using different samples obtained from the same patients, which might include some variation in the viral load contained in the samples. To address this issue, the viral load quantitated using RT-PCR was compared between paired samples; a comparable viral load was observed with a correlation coefficient of 0.8136. Therefore, the equivalent quality of paired samples was verified, and the variation was limited.

## Conclusion

In conclusion, the Lumira Ag test was compared to viral culture, showing high sensitivity and NPV (100% each) relative to viral culture. These findings imply that the Lumira Ag test can be used as a point-of-care test in test-based strategies, allowing social activities to continue during the pandemic.

### Supplementary Information

**Additional file 1.** SARS-CoV-2 isolation from swab sample. (A) Vero E6 cell control and (B) Vero E6 cells inoculated with swab sample showing cytopathic effect (CPE) at 4 days post inoculation. Magnification is 10x for all images.

**Additional file 2. **The results of Lumira Ag testing, viral culture, and RT-PCR testing shown in copies/test.

**Additional file 3. **The viral load of the paired samples as determined by RT-PCR. The X-axis shows the viral load of the samples stored in virus transport media (VTM) used for RT-PCR and viral culture. The Y axis shows the viral load of the samples used for Lumira Ag testing. The viral load was expressed as copies/test. Y = 0.8174x-0.2559, the correlation coefficient was 0.8136.

## Data Availability

The authors confirm that the data supporting the findings of this study are available within the article [and/or] its supplementary materials.

## Abbreviations

COVID-19: Coronavirus disease 2019
Ct: Cycle threshold
ICF: Infected culture fluid
Lumira Ag: LumiraDx SARS-CoV-2 Ag test
NPV: Negative predictive value
PPV: Positive predictive value
VTM: Virus transport media
