# Supplementary material for: The evaluation of a rapid microfluidic immunofluorescence antigen test in detecting the infectiousness of COVID-19 patients
Source: BMC Infect Dis. 2023 Nov 23;23:823. doi: 10.1186/s12879-023-08821-9 (PMC10668452; doi:10.1186/s12879-023-08821-9)
Supplement: Supplementary file 1 — Additional file 1. SARS-CoV-2 isolation from swab sample. (A) Vero E6 cell control and (B) Vero E6 cells inoculated with swab sample showing cytopathic effect (CPE) at 4 days post inoculation. Magnification is 10x for all images. [file 12879_2023_8821_MOESM1_ESM.pptx]

## Slide 1
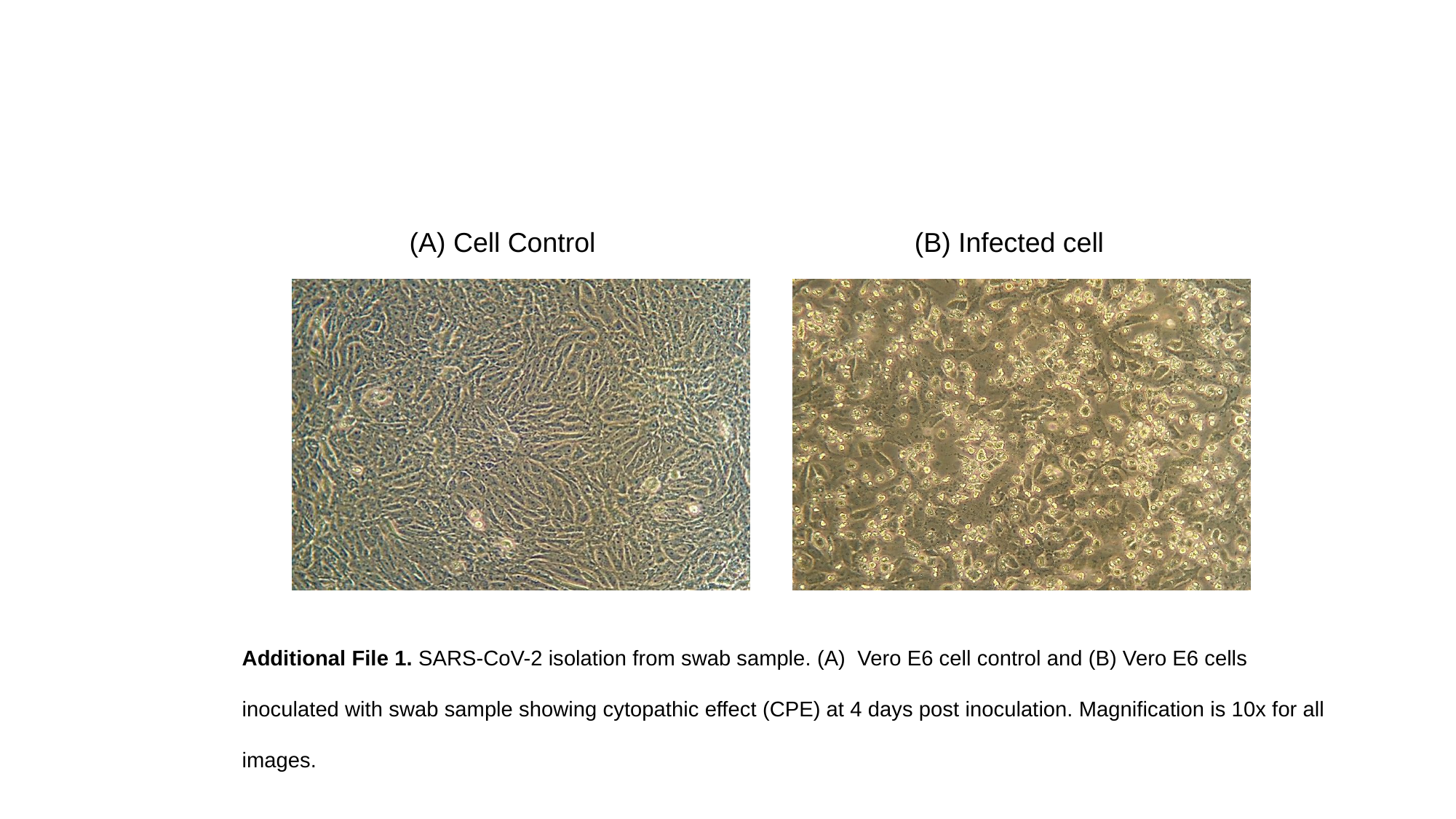

(A) Cell Control
(B) Infected cell
Additional File 1. SARS-CoV-2 isolation from swab sample. (A) Vero E6 cell control and (B) Vero E6 cells inoculated with swab sample showing cytopathic effect (CPE) at 4 days post inoculation. Magnification is 10x for all images.
