# Supplementary material for: The evaluation of a rapid microfluidic immunofluorescence antigen test in detecting the infectiousness of COVID-19 patients
Source: BMC Infect Dis. 2023 Nov 23;23:823. doi: 10.1186/s12879-023-08821-9 (PMC10668452; doi:10.1186/s12879-023-08821-9)
Supplement: Supplementary file 2 — Additional file 2. The results of Lumira Ag testing, viral culture, and RT-PCR testing shown in copies/test. [file 12879_2023_8821_MOESM2_ESM.pptx]

## Slide 1
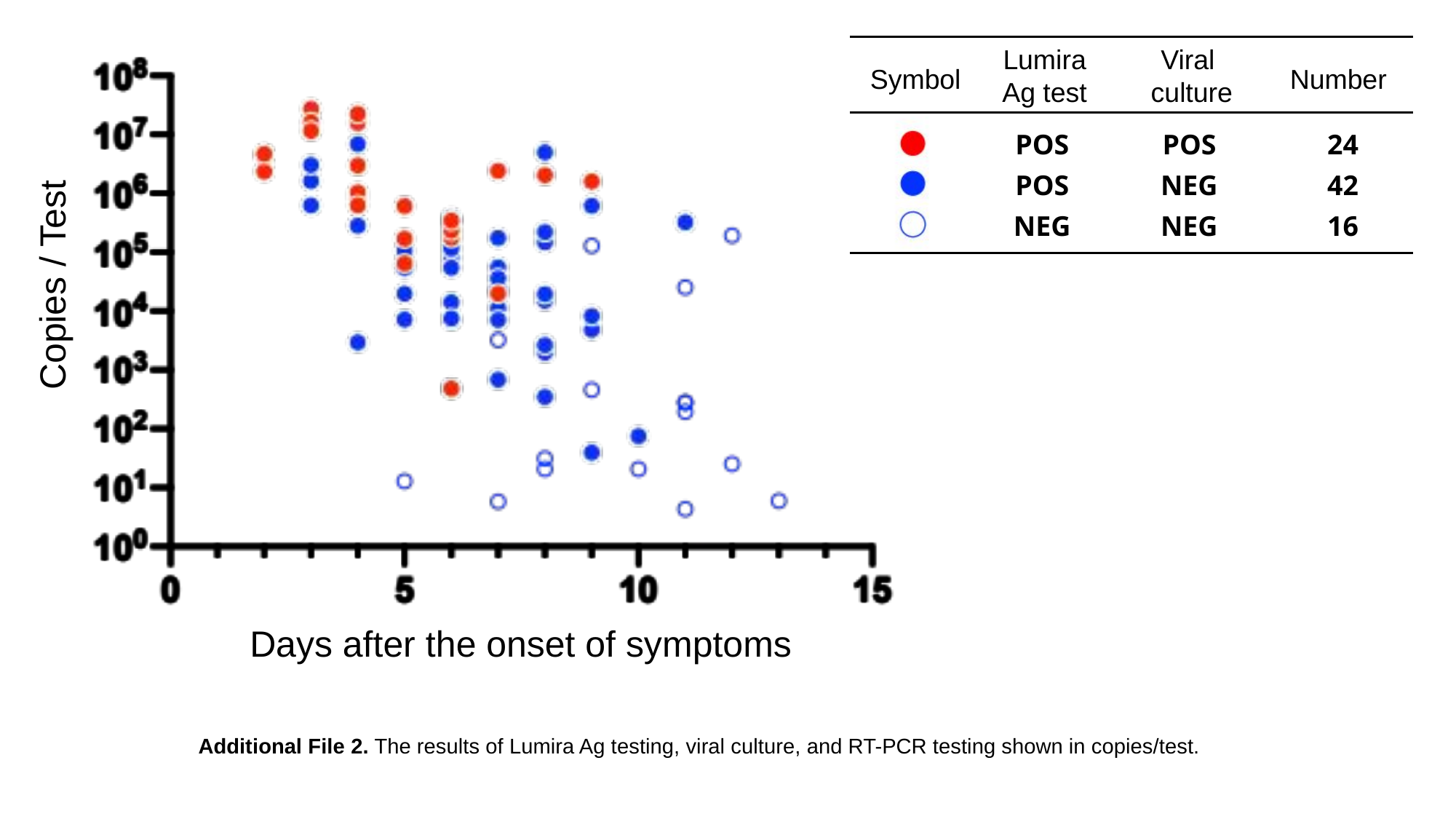

Viral
culture
Lumira
Ag test
Symbol
Number
POS
POS
24
POS
NEG
42
NEG
NEG
16
Copies / Test
Days after the onset of symptoms
Additional File 2. The results of Lumira Ag testing, viral culture, and RT-PCR testing shown in copies/test.
