# Supplementary material for: The evaluation of a rapid microfluidic immunofluorescence antigen test in detecting the infectiousness of COVID-19 patients
Source: BMC Infect Dis. 2023 Nov 23;23:823. doi: 10.1186/s12879-023-08821-9 (PMC10668452; doi:10.1186/s12879-023-08821-9)
Supplement: Supplementary file 3 — Additional file 3. The viral load of the paired samples as determined by RT-PCR. The X-axis shows the viral load of the samples stored in virus transport media (VTM) used for RT-PCR and viral culture. The Y axis shows the viral load of the samples used for Lumira Ag testing. The viral load was expressed as copies/test. Y = 0.8174x-0.2559, the correlation coefficient was 0.8136. [file 12879_2023_8821_MOESM3_ESM.pptx]

## Slide 1
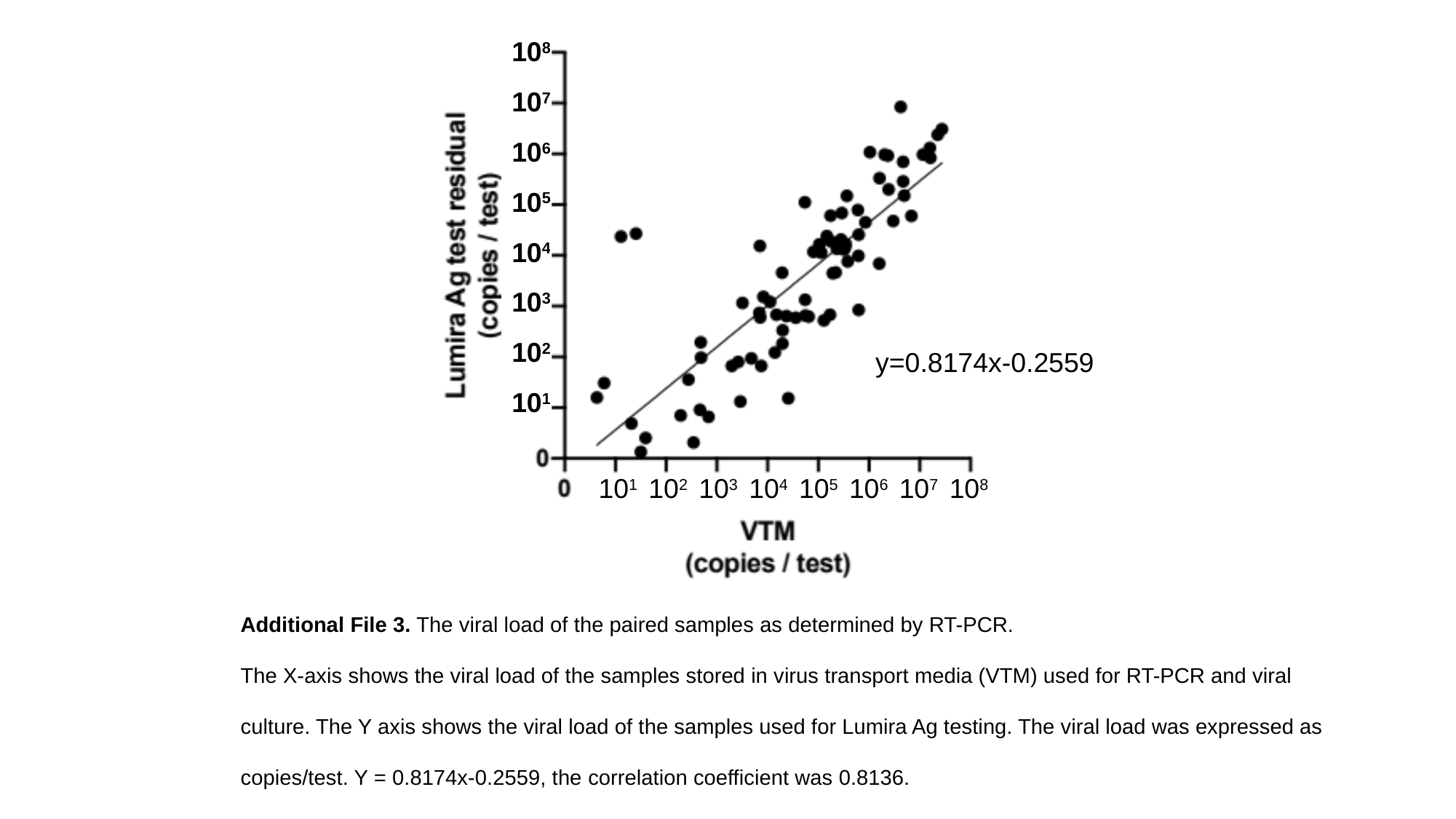

| 108 |
| --- |
| 107 |
| 106 |
| 105 |
| 104 |
| 103 |
| 102 |
| 101 |
y=0.8174x-0.2559
| 101 | 102 | 103 | 104 | 105 | 106 | 107 | 108 |
| --- | --- | --- | --- | --- | --- | --- | --- |
Additional File 3. The viral load of the paired samples as determined by RT-PCR.
The X-axis shows the viral load of the samples stored in virus transport media (VTM) used for RT-PCR and viral culture. The Y axis shows the viral load of the samples used for Lumira Ag testing. The viral load was expressed as copies/test. Y = 0.8174x-0.2559, the correlation coefficient was 0.8136.
